# Society Meetings

**Published:** 1914-04

**Authors:** 


### SOCIETY MEETINGS.

*Brief reports and announcements of meetings of societies of Western New York and those of general scope are requested from Secretaries. Copy should be on hand the fifteenth of each month. Full transactions will be published at cost of composition.*

The Roswell Park Medical Club of Buffalo celebrated its twenty-fifth anniversary on February 9th. Dr. J. H. Potter, the President, gave a short history of the organization, he being one of the original two founders. Dr. J. C. Thompson, the other, acted as toastmaster. The feature of the evening was the presentation to Dr. Park of a handsome hand illumined memento, upon which was inscribed, "Lest men forget the love and esteem in which the members of the Roswell Park Medical Club hold him, whose honored name they have borne for the past twenty-five years, a faithful friend, a brother in good fellowship, an exemplar of professional conduct, a wise counsellor in time of need, they tender this token of their good will."

Then followed five quotations from his own writings. In responding, Dr. Park spoke with the deepest feeling of the friendship and pleasant relations which had so long existed between himself and the members of the club. He paid a touching tribute to the memory of one of the members, Dr. F. C. Busch, recently deceased, and referred in an almost prophetic manner to the next depletion of the number by death. Six days after he himself was called to the great beyond.

The Medical Society of the County of Monroe held a regular meeting on March 17, Dr. Albert C. Snell, the newly elected president being in the chair. The program consisted of a paper by Dr. Roger S. Morris of Clifton Springs Sanitarium on "Gastric Anacidity and Diarrhœa."

THE ROCHESTER PATHOLOGICAL SOCIETY held its meeting on Friday, March 13. Dr. Robert T. Morris of New York reading on the subject: "Let us Settle the Loose Kidney Question."

Further programs as follows: March 19, R. R. Fitch of Rochester; April 2, Allen K. Krause of Saranac Lake; April 16, Paul O. Luedeke of Rochester.

---

THE ROCHESTER ACADEMY OF MEDICINE, Section III, met on March 11 at the Academy rooms, Dr. Joseph Roby reading on "The Infants' Summer Hospital—Some Pediatric Cases."

---

THE HOSPITAL MEDICAL SOCIETY OF ROCHESTER has the following programs, past and future: March 12, "Overfeeding in the Treatment of Digestive Ailments," C. R. Witherspoon; March 26, "Headache," L. W. Jones; April 9, "Local Anæsthesia," C. W. Hoyt; April 23, "New Growth of the Mediastinum," with report of two cases, F. W. Seymour.

---

THE BUFFALO ACADEMY OF MEDICINE has held the following meetings:

February 24—Pathology; The Income Tax as it Affects the Physician, Charles C. Page, Esq., of Buffalo; Certain Immunilogical Reactions and Their Practical Application in the Diagnosis and Treatment of Infections, Dr. Fred C. Bowman of Buffalo.

March 3—Surgery; Intestinal Obstruction, illustrated by stereopticon, Dr. Alexander Primrose of Toronto.

March 10—Medicine; The Burden of Mental Defect, Dr. H. G. Matzinger; Demonstration of Tests to Establish Mental Age of Children, Dr. F. W. Barrows of Buffalo.

March 10—By special invitation, the Academy met in Alumni Hall, University of Buffalo, to hear Prof. Gustav Monod of Vichy and Paris, on "Some Considerations of Enteroptosis," an exposition of Glenard's studies. Dr. Monod is the representative of the French government, touring America in the interests of French post-graduate medical schools. He spoke very flatteringly of American medical colleges, as explaining the falling off in the attendance of American students in France.

March 17—Obstetrics and Gynæcology; Treatment, Operative and Non-operative, or Severe Prolapsus Uteri; Dr. Harry Sturgeon Crossen of St. Louis.

---

THE MEDICAL SOCIETY OF THE COUNTY OF CHEMUNG met in Elmira, March 17. The program was as follows: Malignant Tumors Affecting the Eyeball, Dr. G. M. Case; Treatment of

Acute Alcoholism, Dr. W. C. Byrne; Physio-chemic Principles in Therapy, Dr. L. D. Mottram.

---

The Elmira Academy of Medicine met March 4. The program was as follows: Present Status of the Cure of Cervical Cancer, illustrated by lantern slides, Dr. James E. King of Buffalo; Modern Ophthalmology and Otolaryngology, Dr. G. M. Case of Elmira.

---

The Medical Society of the County of Niagara met in Lockport March 4, 1914. Dr. Wm. G. Bissell of Buffalo demonstrated the Wassermann test. Various committees were appointed. The next meeting will be held in North Tonawanda.

---

At the monthly meeting of Tonawanda Academy of Medicine, held in March, at the residence of Dr. Britt, the paper of the evening on "Vaginal Cæsarean Section" was read by Dr. Irving W. Potter of Buffalo.

---

The Annual Smoker of the I. C. I. Chapter of Nu Sigma Nu was held at the chapter house March 9.

